# Addition of Pulsed Radiofrequency to Suprascapular Nerve Block with Glenohumeral Steroid Injection in Patients with Chronic Shoulder Pain

**DOI:** 10.5812/aapm-164280

**Published:** 2025-09-07

**Authors:** Amr Abdelfatah Sayed, Nahla Yahia Fahmy Kandeel, Mostafa G. Mahran, John Nader Naseef, Azza M. Youssef

**Affiliations:** 1 Department of Anesthesiology, ICU, and Pain Management, Ain Shams University, Cairo, Egypt

**Keywords:** Pulsed Radiofrequency, Suprascapular Nerve Block, Glenohumeral Steroid Injection, Chronic Shoulder Pain

## Abstract

**Background:**

Chronic shoulder pain frequently affects individuals in contemporary societies. However, the benefits of conventional therapies are limited and do not result in sustained clinical improvement.

**Objectives:**

The purpose of this study is to evaluate the potential benefits of integrating pulsed radiofrequency (PRF) neuromodulation into current approaches for managing long-term shoulder pain.

**Methods:**

In this prospective, randomized, clinical interventional study, 60 patients suffering from chronic shoulder pain were randomly assigned to two groups. Group A consisted of 30 patients and received a glenohumeral steroid injection of Diprofos^®^ (betamethasone dipropionate 14 mg/2 mL) along with an ultrasound-guided suprascapular nerve (US SSN) block using 10 mL of 0.25% preservative-free bupivacaine. Group B, also comprising 30 patients, underwent the same protocol as group A, with the additional administration of PRF treatment targeting the suprascapular nerve (SSN). Pain and function were assessed using the Shoulder Pain and Disability Index (SPADI), Numerical Rating Scale (NRS), and shoulder active range of motion (AROM) at baseline, 15 days, 1 month, 3 months, and 6 months post-procedure.

**Results:**

Both groups showed significant pain and functional improvement throughout the study course. However, group B exhibited superior outcomes at all follow-up points. The SPADI score decreased from 69.71 ± 16.54 at baseline to 48.16 ± 15.77 at six months in group B, compared to 72.02 ± 14.51 to 64.91 ± 14.34 in group A (P < 0.001). Median NRS scores at six months were also significantly lower (P < 0.0001) in group B [2.00 (IQR: 1.00 - 4.00)] compared to group A [5.00 (IQR: 4.00 - 6.00)]. The AROM measurements at six months favored group B for internal rotation (74.77 ± 6.84 vs. 67.10 ± 9.26, P = 0.001), external rotation (74.40 ± 9.90 vs. 67.23 ± 9.32, P = 0.006), and abduction (163.23 ± 9.96 vs. 155.30 ± 10.13, P = 0.003). Flexion improvement at six months was greater in group B (162.80 ± 21.14) than in group A (154.93 ± 12.76), though not statistically significant (P = 0.086).

**Conclusions:**

This study showed that the addition of PRF therapy to the SSN significantly improved clinical outcomes compared to the suprascapular nerve block (SSNB) combined with intra-articular corticosteroid injection in patients with chronic shoulder impingement syndrome. The combined modality led to significantly better outcomes in terms of pain alleviation, range of motion, and functional capacity.

## 1. Background

Shoulder pain is the second most frequent musculoskeletal complaint in adults. However, it remains commonly under-treated, which accelerates its progression into a chronic condition (1). Therapeutic options for chronic shoulder pain include conservative measures, minimally invasive interventions, and surgical procedures. Conservative therapies include rest, rehabilitation exercises, shock wave therapy, and the administration of analgesics and anti-inflammatory medications (2). Subacromial steroid injection (SSI) is a common intervention that is effective in reducing shoulder pain and improving outcomes (3). Steroid injection has superior analgesic properties compared to conventional therapies, including non-steroidal anti-inflammatory drugs (NSAIDs). However, the benefits of steroid injections are short-term, with symptom relief limited to 4 to 6 weeks only, and may not result in a clinically meaningful improvement (4).

The suprascapular nerve (SSN) arises from the C5 and C6 nerve roots within the upper trunk of the brachial plexus. It supplies sensory innervation to the acromioclavicular and glenohumeral joints, the coracoclavicular ligament, and the subacromial bursa. Functionally, the motor fibers of the SSN supply the supraspinatus and infraspinatus muscles, contributing to the dynamic stability and movement of the shoulder joint (5). Almost 70% of the sensory innervation to the shoulder joint is mediated by the SSN. Therefore, its blockade is considered a potentially effective intervention for managing chronic shoulder pain (6). Despite their therapeutic utility, nerve blocks, like steroid injections, are limited by their temporary duration of action (7).

Pulsed radiofrequency (PRF) therapy is an accepted treatment modality for targeting peripheral nerves due to its extended pain-relieving effects relative to nerve blocks (8). Most studies focused on nerve block or PRF alone, with varying results and follow-up durations. We hypothesized that the addition of PRF to standard treatment protocols for chronic shoulder pain would enhance therapeutic efficacy, leading to greater reductions in pain and improved shoulder joint function.

## 2. Objectives

This study aims to evaluate the potential benefits of adding PRF neuromodulation to suprascapular nerve block (SSNB) and intra-articular betamethasone dipropionate injection for managing long-term shoulder pain due to impingement syndrome.

## 3. Methods

### 3.1. Study Design and Settings

This is a prospective, randomized study conducted between September 2023 and April 2025. It was conducted in the Department of Anesthesiology, Critical Care, and Pain Management at Ain Shams University hospitals. The study protocol was approved by the Institutional Research Ethics Committee of the Faculty of Medicine, Ain Shams University (FMASU MD 128/2023) and prospectively registered (ClinicalTrials.gov Identifier: NCT06106490). Prior to enrollment, all participants provided written informed consent.

### 3.2. Population

A total of 60 patients, aged between 21 and 60 years, categorized as American Society of Anesthesiologists (ASA) physical status I or II, were enrolled. Inclusion criteria included adult patients (≥ 18 years) with chronic shoulder pain due to impingement syndrome for a duration exceeding three months, and patients with insufficient response to conservative therapies and oral analgesics. All patients were diagnosed and referred by medical practitioners, orthopedists, and physiotherapists. Patients were diagnosed with impingement syndrome based on their history, standardized clinical criteria, and imaging results. The clinical diagnosis required at least one positive impingement test (Neer’s sign and/or Hawkins-Kennedy test) combined with confirmatory imaging (ultrasound or MRI).

Exclusion criteria included patients with contraindications to regional anesthesia, such as local infection at the injection site, coagulopathy, or hypersensitivity to any study medication; patients who refused to consent; patients with a history of shoulder surgery, or who underwent other shoulder interventions within three months before the study procedure or one year following it; patients with uncontrolled diabetes mellitus; or chronic pain syndromes secondary to alternative shoulder pathologies, such as fibromyalgia, cervical discopathy, or brachial plexus injury.

### 3.3. Randomization

Participants were randomly allocated to one of two groups through a simple random sampling technique using a computer-generated random number table into two equal groups (n = 30 per group). Group A included patients receiving ultrasound-guided SSNB in addition to intra-articular steroid injection. Group B included patients receiving PRF treatment to the SSN in addition to the same SSNB and intra-articular steroid protocol as group A (Appendix 1 in Supplementary File). Allocation concealment was done using sequentially numbered, opaque, and sealed envelopes prepared by an independent researcher not involved in recruitment or assessment. The envelopes were opened after enrollment to prevent selection bias.

### 3.4. Study Objectives and Outcome Measures

A comprehensive clinical examination and meticulous review of medical history were conducted for all enrolled subjects. Patients were evaluated in the pain clinic at baseline, on day 15, after one month, after three months, and after six months following the intervention. The primary objective was to evaluate pain and functional limitations using the Shoulder Pain and Disability Index (SPADI), which is a self-reported instrument divided into two domains: Pain (five items) and disability (eight items). The disability domain measured the difficulty experienced in daily activities involving upper limb functions. The composite score ranged from 0 to 130, with percentage values interpreted as follows: Zero percent reflected no shoulder disability, and 100% reflected maximum functional impairment (9).

The secondary study outcomes included patient demographics, Numerical Rating Scale (NRS) for pain, and active range of motion (AROM). Patients were instructed on the use of the NRS for pain assessment, wherein they were asked to select a number from 0 to 10, where 0 indicated 'no pain at all' and 10 indicated 'the most severe pain imaginable' (10). A goniometer was utilized to evaluate the AROM of the shoulder joint, providing an objective assessment of functional movement, including flexion, abduction, internal, and external rotation.

### 3.5. Interventions

#### 3.5.1. Preoperative Evaluation and Monitoring

All patients underwent standard preoperative laboratory tests, including complete blood count (CBC), bleeding time, prothrombin time (PT), partial thromboplastin time (PTT), International Normalized Ratio (INR), and other relevant investigations. The interventions were carried out in fully equipped operating rooms, with electrocardiographic monitoring, non-invasive blood pressure assessments, and peripheral oxygen saturation. Peripheral intravenous vascular access was established, with mild sedation using 1 - 2 mg of midazolam. Patients were placed in the sitting position for all interventions. Ultrasound-guided procedures were carried out by the same experienced pain specialist to ensure consistency and procedural accuracy.

#### 3.5.2. Intra-articular Steroid Injection (Group A and B)

Ultrasound-guided intra-articular steroid injections were administered in the lateral decubitus position, with the ipsilateral shoulder oriented upward, and the arm positioned across the chest to rest on the contralateral shoulder. An antiseptic solution was applied to the skin covering the shoulder, subacromial area, and joint space. The tip of the acromion was identified by palpation. The lateral edge of a high-frequency linear ultrasound probe (5 - 13 MHz) was aligned over the acromion tip, and the medial edge was angled approximately 20 degrees medially toward the scapula. Local anesthesia was induced in the posterior aspect of the glenohumeral joint by infiltrating 1% lidocaine. Subsequently, a 22-G Quincke spinal needle was inserted, under ultrasound guidance, 1 cm lateral to the transducer margin and maneuvered medially to laterally using an in-plane approach. After careful aspiration, 2 mL of betamethasone dipropionate (Diprofos^®^) (14 mg) was gently injected.

#### 3.5.3. Ultrasound-Guided Suprascapular Nerve Block (Groups A and B)

Patients were seated with both arms relaxed. The scapular spine was identified by manual palpation. A high-frequency linear transducer (5 - 13 MHz) was positioned parallel to the scapular spine and slowly shifted in a lateral direction until the characteristic “U”-shaped suprascapular notch became visible. The SSN was visualized as a hyperechoic structure situated beneath the transverse scapular ligament within the suprascapular notch. The pulsation of the suprascapular artery was visualized. Then, the 22-gauge Quincke spinal needle was introduced to the suprascapular notch, and 10 mL of 0.25% bupivacaine was injected perineurally under image guidance.

#### 3.5.4. Pulsed Radiofrequency Treatment (Group B Only)

 In group B patients, an additional PRF procedure targeting the SSN was performed. The targeted skin area was disinfected and prepared following aseptic procedural standards. The posterior approach was applied, where a high-frequency linear probe was positioned cephalad and parallel to the scapular spine to locate the suprascapular notch. Within the scapular notch, the SSN appears as a bright, echogenic structure located inferior to the transverse scapular ligament on ultrasound imaging. A 10 cm RF needle with a 10 mm active tip was inserted toward the nerve guided by ultrasound. Both sensory and motor stimulations were used to confirm the nerve location. The PRF was then applied using a straight-tip radiofrequency probe connected to the NeuroTherm 1100 generator at 42°C, with a pulse width of 20 ms, amplitude of 45 V, and frequency of 2 Hz for a total of 480 seconds. Patients were monitored after the procedure and discharged once stable and without complications such as bleeding, pain, or pneumothorax.

### 3.6. Sample Size Calculation and Statistical Analysis

The results of Ergonenc and Beyaz (11) showed that the mean score of the SPADI decreased among patients with chronic shoulder pain who underwent ultrasound-guided suprascapular nerve (US SSN) PRF after 6 months compared to baseline (39.9 ± 10.64 versus 64.95 ± 8.21, respectively). In our study, sample size calculation, performed using MedCalc Software (version 20.18; MedCalc Ltd., Ostend, Belgium), indicated that a minimum of 30 patients per group is required to achieve a statistical power of 0.90 for detecting a SPADI difference of at least 10%, at a significance level of 0.05, assuming a standard deviation of the SPADI score of 11.72%.

Data were collected, coded, tabulated, and then analyzed using the SPSS software package (IBM SPSS Statistics for Windows, Version 22.0. Armonk, NY: IBM Corp., 2013). Numerical variables were presented as mean (standard deviation), and categorical variables were presented as frequency (%). Comparisons of numerical variables were done using an unpaired *t*-test, while Fisher’s exact test was used for comparisons of nominal variables. Any difference with a P-value < 0.05 is considered statistically significant.

## 4. Results

In this randomized clinical trial, demographic data in terms of age and weight were comparable between both groups (P > 0.05 for all, Table 1). Although there was a female predominance in group A, this was reversed in group B; however, the difference was statistically insignificant (P > 0.073).

**Table 1.** Baseline Demographic Characteristics of Study Participants ^a^

Demographic Data	Group A (N = 30)	Group B (N = 30)	P-Value
**Age (y)**	43.53 ± 10.65	43.50 ± 8.95	0.99 ^b^
**Weight (kg)**	78.83 ± 12.05	80.03 ± 10.72	0.685 ^b^
**Sex**			0.073 ^c^
Male	12 (40)	19 (63.33)	
Female	18 (60)	11 (36.67)	

^a^ Values are expressed as mean ± SD or No. (%).

^b^ Student *t*-test.

^c^ Chi-square.

The two groups were compared in terms of SPADI scores at multiple points. At baseline, there was no significant difference between the two groups (P = 0.568). However, group B showed more improvement in shoulder function at day 15 (P = 0.010) and in the following months (P < 0.001, Table 2). 

**Table 2.** Comparison of Shoulder Pain and Disability Index Scores Between Group A and Group B at Baseline and Follow-up Timepoints ^a^

SPADI Score	Mean ± Standard Deviation	Mean Difference	95% Confidence Interval of the Difference		P-Value
			Lower	Upper	
**At baseline**		2.3100	-5.7318	10.3518	0.568
Group A	72.02 ± 14.51				
Group B	69.71 ± 16.54				
**After 15 days**		11.4663	2.8674	20.0652	0.010
Group A	57.07 ± 15.99				
Group B	45.60 ± 17.25				
**After one month**		15.7430	7.9090	23.5770	< 0.001
Group A	57.49 ± 13.98				
Group B	41.75 ± 16.25				
**After three months**		17.2797	9.3031	25.2562	< 0.001
Group A	61.59 ± 15.52				
Group B	44.31 ± 15.35				
**After six months**		16.7463	8.9557	24.5369	< 0.001
Group A	64.91 ± 14.34				
Group B	48.16 ± 15.77				

Abbreviation: SPADI, Shoulder Pain and Disability Index.

^a^ Values are expressed as mean ± SD.

**Figure 1.**
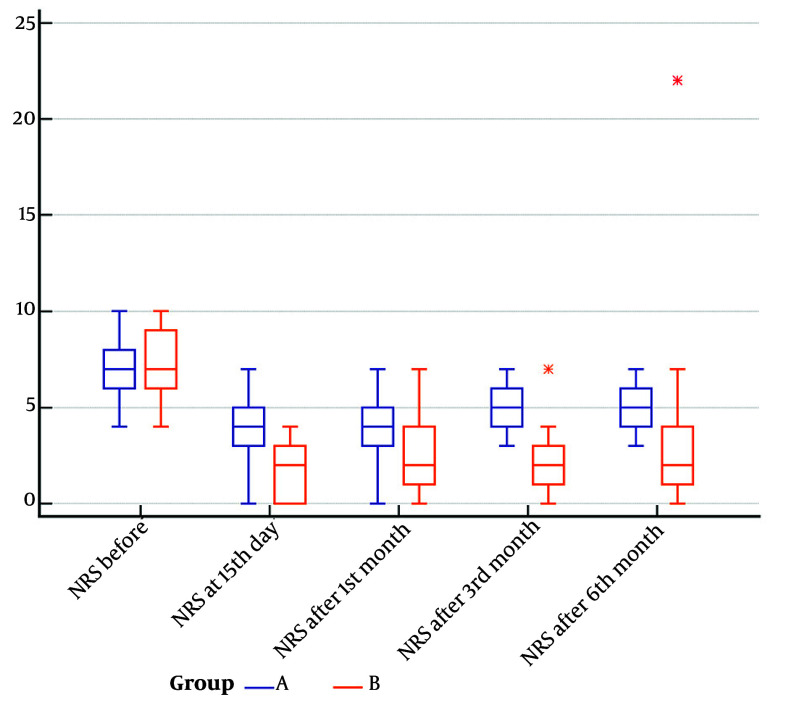
Numeric Rating Scale (NRS) pain scores of group A and group B at baseline and follow-up timepoints

The NRS scores were compared in both groups at baseline and all timepoints (Figure 1). At baseline, both groups had comparable median pain levels (group A: 7.00, range: 6.00 - 8.00; group B: 7.00, IQR: 6.00 - 9.00; P = 0.6190). After 15 days, group B showed significantly lower pain scores compared to group A (group A: 4.00, IQR: 3.00 - 5.00; group B: 2.00, range: 0.00 - 3.00; P = 0.0003). The median NRS remained significantly lower in group B after one month (group A: 4.00, IQR: 3.00 - 5.00; group B: 2.00, range: 1.00 - 4.00; P = 0.0001), three months (group A: 5.00, IQR: 4.00 - 6.00; group B: 2.00, range: 1.00 - 3.00; P < 0.0001), and six months (group A: 5.00, IQR: 4.00 - 6.00; group B: 2.00, range: 1.00 - 4.00; P < 0.0001).

Based on the AROM measurements at day 15, group B showed significantly greater improvement in shoulder flexion (P = 0.022), internal rotation (P = 0.016), and external rotation (P = 0.004) compared to group A; however, there was no significant difference between the two groups in abduction (P = 0.373). After one month, group B showed significantly better improvement in all shoulder AROM measures compared to group A, including flexion (P = 0.009), internal rotation (P = 0.015), external rotation (P = 0.0095), and abduction (P = 0.006). At three months, group B showed significantly better shoulder mobility than group A across all AROM measures, including flexion (P = 0.002), internal rotation (P = 0.005), external rotation (P = 0.015), and abduction (P = 0.0001). At six months, group B showed significantly greater improvement in shoulder internal rotation (P = 0.001), external rotation (P = 0.006), and abduction (P = 0.003) compared to group A, while the difference in flexion was not statistically significant (P = 0.086, Table 3). 

**Table 3.** Comparison of Shoulder Active Range of Motion Between Group A and Group B at All Timepoints ^a^

Variables	Group A (N = 30)	Group B (N = 30)	P-Value ^b^
**AROM at day 15**			
AROM flexion (degree)	156.83 ± 15.11	165.00 ± 11.60	0.022
AROM internal rotation (degree)	66.50 ± 11.08	73.00 ± 9.06	0.016
AROM external rotation (degree)	66.73 ± 10.01	74.73 ± 10.83	0.004
AROM abduction	155.40 ± 10.87	159.10 ± 19.80	0.373
**AROM after one month **			
AROM flexion (degree)	158.20 ± 12.75	166.33 ± 10.25	0.009
AROM internal rotation (degree)	70.40 ± 10.42	76.67 ± 8.94	0.015
AROM external rotation (degree)	69.20 ± 10.37	76.53 ± 10.84	0.0095
AROM abduction	157.50 ± 9.98	165.03 ± 10.60	0.006
**AROM after three months **			
AROM flexion (degree)	153.20 ± 11.92	166.07 ± 10.60	0.002
AROM internal rotation (degree)	69.00 ± 9.96	75.83 ± 8.01	0.005
AROM external rotation (degree)	68.73 ± 10.33	75.40 ± 10.36	0.015
AROM abduction (degree)	155.63 ± 10.98	166.13 ± 8.94	0.0001
**AROM after six months **			
AROM flexion (degree)	154.93 ± 12.76	162.80 ± 21.14	0.086
AROM internal rotation (degree)	67.10 ± 9.26	74.77 ± 6.84	0.001
AROM external rotation (degree)	67.23 ± 9.32	74.40 ± 9.90	0.006
AROM abduction (degree)	155.30 ± 10.13	163.23 ± 9.96	0.003

Abbreviation: AROM, active range of motion.

^a^ Values are expressed as mean ± SD or median and IQR.

^b^ Student *t*-test.

**Figure 2.**
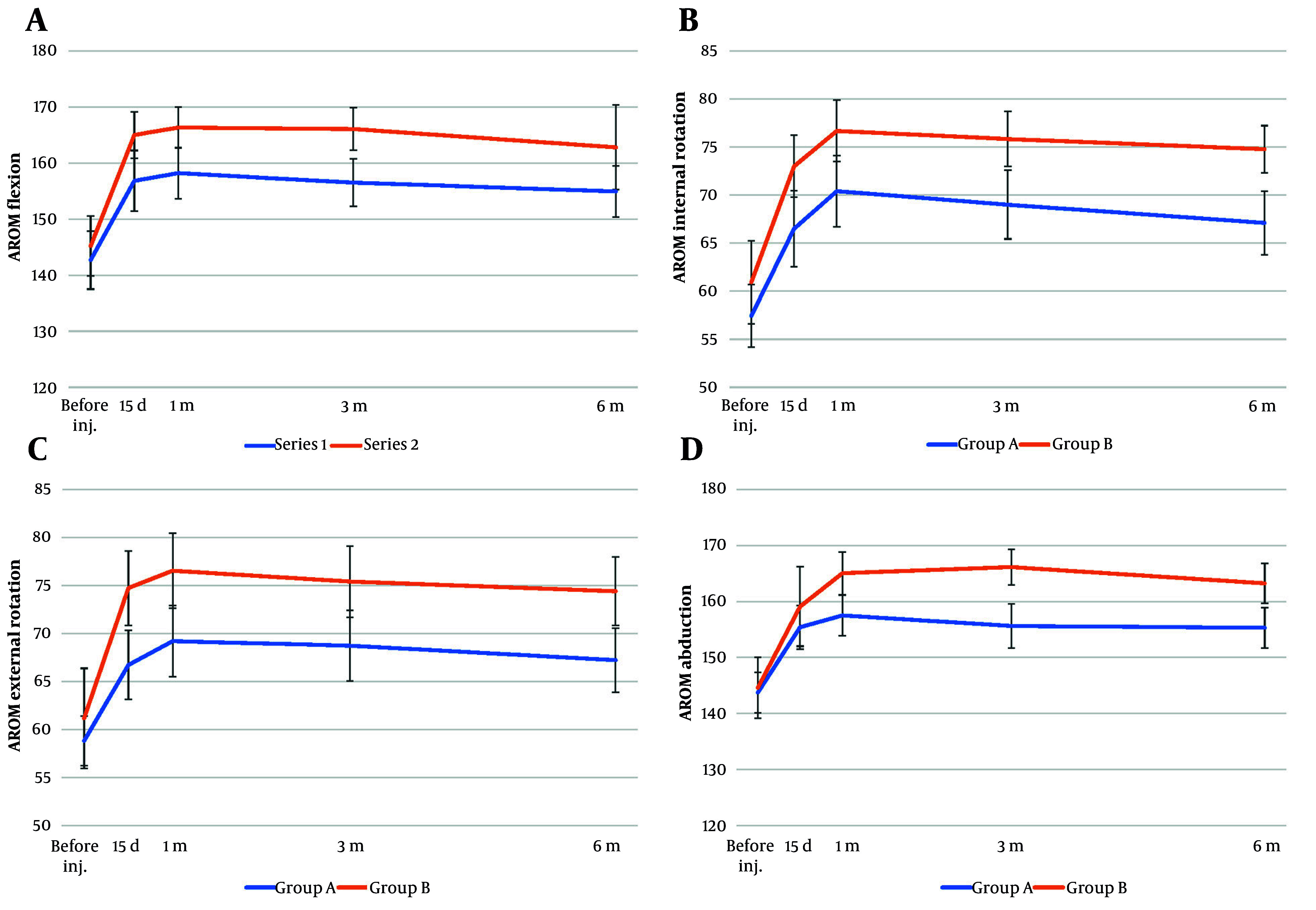
Comparison of shoulder active range of motion (AROM) measurements over time between group A (blue line) and group B (orange line) at baseline (before injection), 15 days, 1 month, 3 months, and 6 months post-procedure: A, AROM in flexion (degrees); B, AROM in internal rotation (degrees); C, AROM in external rotation (degrees); D, AROM in abduction (degrees; error bars represent standard deviations).

Figure 2 shows that group B consistently achieved greater and more sustained improvements in shoulder AROM across all motion dimensions, including flexion, internal rotation, external rotation, and abduction, compared to group A from day 15 to six months post-intervention. Group A showed slight improvements that either plateaued or declined over time. In addition, no treatment-related adverse events or complications were observed in either group during the procedure or throughout the follow-up period of the study.

## 5. Discussion

In this study, we evaluated the therapeutic effect of adding PRF to SSNB combined with ultrasound-guided intra-articular steroid injection in chronic shoulder pain patients with impingement syndrome. The study focused on changes in pain and function scores in patients with shoulder pain for at least three months with no response to conservative therapies. Both groups exhibited an improvement in pain scores following intervention. However, our findings support the hypothesis that adding PRF to SSNB and intra-articular steroids significantly enhances pain relief and shoulder function in patients with chronic impingement syndrome. Patients who received additional PRF had greater reductions in SPADI scores compared to the injection-only group throughout the six-month study duration. In addition, they reported consistently lower numeric pain scores starting from day 15 onward, indicating faster and sustained pain relief.

Regarding shoulder motion, patients receiving adjuvant PRF maintained better shoulder AROM in most planes, suggesting that PRF could prevent the decline in shoulder motion. Both groups in our study demonstrated a reduction in SPADI and NRS scores. This confirms the efficacy of SSNB combined with intra-articular corticosteroids, with or without PRF, in managing patients with chronic shoulder pain (12). However, patients receiving additional PRF treatment showed greater and consistent improvements in pain reduction, shoulder mobility, and overall function starting from day 15 and throughout the 6-month study follow-up period. This aligns with the existing literature that suggests PRF as an effective adjunct therapy for enhancing pain relief and functional recovery in chronic shoulder pain secondary to shoulder impingement syndrome (13-15).

Bergamaschi et al. found that ultrasound-guided PRF of the SSN resulted in greater pain relief compared to nerve block alone in patients with chronic shoulder pain. The PRF-treated group had significantly reduced pain during shoulder movement in the weeks following the procedure; however, that study reported no difference in shoulder range of motion between the two groups at the third month (12). In contrast, our study showed early AROM advantages in the PRF group starting as early as 15 days post-intervention. This discrepancy could be due to the inclusion of intra-articular steroids in both groups.

Our study design is novel in evaluating PRF in combination with SSNB and intra-articular steroids. Few previous studies exist; however, our study differs from earlier research in study design, PRF duration, and the type of corticosteroid used. Cetingok and Serce (16) observed significant pain relief and functional improvements following combined PRF, SSNB, and intra-articular steroid treatments. Similar to our study, patients receiving this regimen had lower NRS scores, longer durations of pain relief, and better overall patient satisfaction (P < 0.05). However, the PRF duration (240 seconds) in their study was shorter than in our study (480 seconds). This suggests that adjunctive therapies may compensate for shorter neuromodulation periods and enhance pain relief in terms of intensity and duration.

Liliang et al. (15) suggested the clinical efficacy of PRF alone, applied for 180 seconds, showing significant reductions in Visual Analog Scale (VAS) and SPADI scores at one and six months (P < 0.001). Their study suggested that even shorter PRF durations are beneficial in reducing pain and disability. Our study supported the role of PRF in providing pain and function improvement compared to the clinical benefit without PRF. These benefits were also demonstrated by Luleci et al. (14), who observed significant pain reduction (P < 0.001) and functional improvement (P < 0.001) after three and six months of PRF treatment (480 seconds). The percentage of patients with sustained pain reduction was higher after six months of PRF treatment in Luleci et al.’s study (78.9%) compared to Liliang et al.’s study (69.2%) 15. These findings could support the advantages of longer PRF durations, especially when combined with nerve blocks and intra-articular steroid injections (14).

Other studies supported the clinical benefits of PRF lesioning of the SSN combined with physical therapy, compared to physical therapy alone. Wu et al. (13) found a rapid (6.1 ± 3.4 vs. 28.1 ± 9.2 days; P < 0.001) and substantial pain relief (reduction of VAS score: 40% vs. 4.7%; P < 0.001) when combining PRF (180 seconds) with physical therapy in patients with adhesive capsulitis. Additionally, the VAS, SPADI, and Passive Range of Motion (PROM) scores improved significantly in patients receiving the PRF and physical therapy combination (P < 0.05). Together, our study and Wu et al.’s findings suggest that the analgesic and functional benefits of PRF in chronic shoulder pain persist for at least six months (13). Prolonged pain relief allows patients to undergo physiotherapy with minimal discomfort, resulting in accelerated functional recovery (17). In contrast, other studies questioned the role of PRF in improving pain and function in patients with chronic pain. The randomized controlled study by Youssef et al. (18) found more rapid and sustained pain relief with intra-articular triamcinolone acetonide injections alone compared to PRF (240 seconds), until three months post-intervention (P = 0.018). However, there was no observed difference in the VAS score of both groups after 6 months of follow-up (P = 0.096). This could suggest that intra-articular steroid injection may offer superior therapeutic benefits over PRF in select patient populations. However, the variability in responses could differ between diverse patient populations or procedural specifics.

Differences in PRF duration, type of corticosteroid injection, selected local anesthetic, and adjunctive therapies are considerable variables that can impact treatment outcomes. For instance, the selection of betamethasone dipropionate in our study may have influenced analgesic outcomes due to its pharmacokinetic profile and safety advantages, such as smaller particulate size and reduced embolization risk compared to triamcinolone. Our study also used bupivacaine, a long-acting amide anesthetic with a slow onset and a prolonged duration of action, compared to lidocaine (19).

The meta-analysis conducted by Pushparaj et al. (20) also suggested insufficient evidence supporting PRF efficacy; however, the reliability of this review remains limited due to certain methodological concerns, such as the inclusion of observational studies, case series, and case reports, the heterogeneity in study designs, variable participant characteristics, different follow-up intervals, and inconsistent treatment protocols.

Sinha et al. observed that ultrasound-guided PRF of the SSN combined with intra-articular dexamethasone injection was an effective treatment for the management of chronic shoulder pain. The PRF significantly improved the AROM in all directions, including flexion, extension, adduction, abduction, external rotation, and internal rotation, with a significant increase (P < 0.001) after the intervention (17). Abo Elfadl et al. also found that shoulder mobility significantly improved after PRF, where the functional range, using the simple shoulder test (SST), improved from 6.5% at baseline to 76.5% post-PRF at 12 weeks of treatment (21).

Similarly, our trial found that patients receiving adjunct PRF had significantly better AROM and SPADI scores up to six months of follow-up, suggesting that patients were better at performing exercises and daily tasks. However, the slight decline in AROM flexion in group B, observed after 6 months, could result from a possible fading of the pharmacological and neuromodulatory effects of the intervention. Our study’s positive outcomes highlight the potential value of incorporating PRF treatment of the SSN at 42°C for 480 seconds in enhancing the therapeutic benefit for patients with chronic shoulder impingement syndrome, compared to the use of SSN block and intra-articular steroid injections alone.

Additionally, the unique mechanism of PRF neuromodulation, without nerve destruction, likely contributed to sustained therapeutic outcomes, which could improve patient satisfaction and participation in rehabilitation activities (13). In real-world settings, this treatment strategy offers a minimally invasive, outpatient procedure that could reduce the need for more invasive surgeries. We also believe that better improvement of pain control and mobility with this combination may result in better engagement in rehabilitation programs and longer functional recovery. Future research should assess the cost-effectiveness of this combination management and identify patient subgroups that could most likely benefit from it in real-world practice.

### 5.1. Conclusions

In conclusion, our findings support the incorporation of PRF into standard treatment protocols for chronic shoulder pain secondary to impingement syndrome, providing sustained pain relief, improved shoulder mobility, and enhanced patient comfort. Given its favorable risk-benefit profile, PRF represents a promising adjunct therapy, warranting further investigation to optimize protocols and confirm long-term benefits. Further studies with larger, multi-center cohorts, longer follow-up durations, and randomized-controlled comparisons are recommended to assess the long-term effectiveness of management modalities.

### 5.2. Limitations

This study has several limitations that warrant consideration. First, the relatively small sample size may limit the statistical power and reduce the generalizability of the findings. Additionally, being a single-center study, the results may be influenced by local clinical practices or population characteristics, which could affect external validity. The absence of a placebo or sham-control group further limits the ability to attribute improvements solely to the PRF intervention. Placebo effects, including benefits from procedural elements such as needle placement, local anesthetic administration, or patient expectations, could not be ruled out.

Moreover, the use of subjective outcome measures such as the Visual Numerical Rating Scale (VNRS) and the Modified MacNab criteria, while validated, may be influenced by individual perceptions and expectations. The lack of blinding in both patients and assessors also raises the risk of performance and detection bias. Furthermore, the follow-up duration was limited to six months, which restricts insights into the long-term sustainability of pain relief and functional improvement. Lastly, variations in patient characteristics, such as chronicity, underlying etiology of shoulder pain, and previous treatments, may introduce heterogeneity that could affect the interpretation of outcomes.

aapm-15-5-164280-s001.pdf

## Data Availability

The authors confirm that the data supporting the findings of this study are available within the article.

## Clinical Trial Registration Code:

NCT06106490.
