# Supplementary figures and images for: Addition of Pulsed Radiofrequency to Suprascapular Nerve Block with Glenohumeral Steroid Injection in Patients with Chronic Shoulder Pain
Source: Anesth Pain Med. 2025 Sep 7;15(5):e164280. doi: 10.5812/aapm-164280 (PMC12523909; doi:10.5812/aapm-164280)

## Appendix 1. Flow Diagram of Patient Enrollment and Allocation

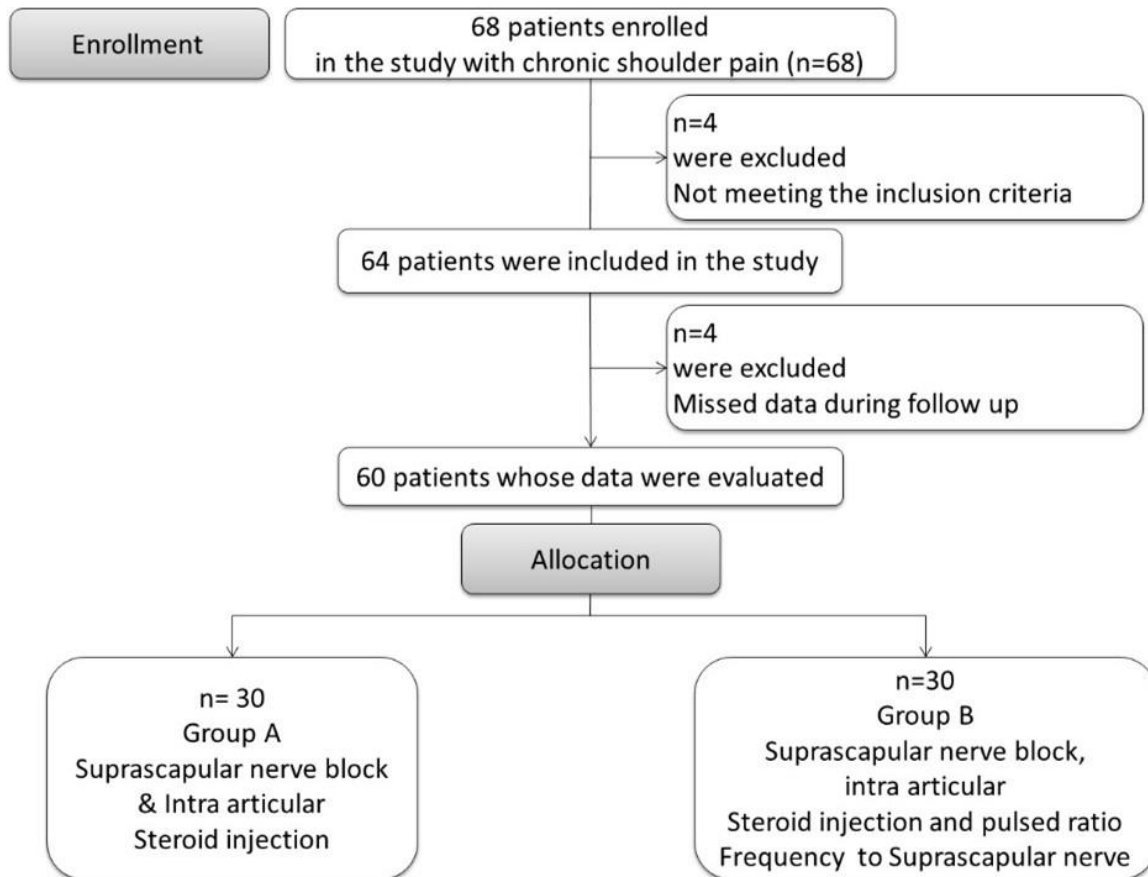

Supplement: aapm-15-5-164280-s001.pdf [file aapm-15-5-164280-s001.pdf]
